# Editorial: Water use efficiency (WUE): enhancing crop resilience using agronomic and genetic strategies under abiotic stress conditions

**DOI:** 10.3389/fpls.2026.1951554

**Published:** 2026-08-06

**Authors:** Mallesham Bulle, Papa Rao Vaikuntapu, Prasanta K. Subudhi

**Affiliations:** 1 School of Plant, Environmental and Soil Sciences, Louisiana State University Agricultural Center, Baton Rouge, LA, United States; 2 ICAR-Indian Institute of Rice Research, Hyderabad, India

**Keywords:** agronomic management, crop resilience, drought tolerance, precision irrigation, water use efficiency

## Introduction

The global population is projected to reach 9.1 billion by 2050, requiring a 70% increase in food production to meet rising demand (FAO, 2009). At the same time, projected increases in global temperature of 1–2.5 °C over the next 50 years, altered precipitation patterns, and more frequent drought and heat-stress events are expected to modify vapor pressure deficits, soil water availability, and crop water-use dynamics, thereby constraining plant productivity (Lesk et al., 2022). Because agriculture accounts for approximately 70% of global freshwater consumption, elucidating plant–soil–atmosphere interactions remains fundamental to advancing water-use efficiency (WUE) research.

Maximizing WUE, defined as yield per unit of water used, requires an integrated approach that combines agronomic, physiological, biotechnological, and engineering strategies. Achieving this goal depends on the aligning field-level water and nutrient management practices with the genetic determinants that regulate water acquisition, transport, and conservation in crop species (Ghannoum et al., 2025). Accordingly, this Research Topic was initiated to highlight this convergence. The five contributions address irrigation scheduling, genotype-by-management interactions under salinity, comparative physiology of water-conserving genotypes, and genetic dissection of stress tolerance in rice and *Brassica juncea*.

From an agronomic perspective, Bai et al. examined irrigation and fertilization scheduling in fruit trees. Their two-year field study evaluated sand tube irrigation (STI) combined with different irrigation and nitrogen levels to improve jujube production in arid regions. Compared with conventional surface drip irrigation, STI increased jujube yield by 15–18% and improved average net income by 51%. By combining regression analysis with an entropy-weighted TOPSIS framework, the study identified 3,600 m^3^ ha^-1^ irrigation and 370 kg N ha^-1^ nitrogen as the optimal combination. Overall, this approach offers practical guidance for improving water and nitrogen management in arid orchard systems.

Salinity compounds water stress in many irrigated regions. Bekmurodov et al. addressed this challenge by evaluating a sustainable intensification strategy for fine-staple cotton (*Gossypium barbadense*) grown on saline soils in Uzbekistan. An intensified irrigation regime, combined with a salt-tolerant genotype and high-density twin-row planting, increased seed cotton yield by 32% and WUE by 25% compared with conventional management. The approach also slowed the decline in soil porosity and stabilized the root-zone salt balance. These findings are consistent with recent work in *Gossypium hirsutum*, where water stress induced the preliminary expression of C4 photosynthetic traits, higher vein density, and increased phosphoenolpyruvate carboxykinase (PEPC) activity, thereby improving leaf WUE (Lei et al., 2025). Together, these results indicate that integrating salt-tolerant genotypes with optimized irrigation and high-density planting can improve cotton productivity and water efficiency under saline conditions, although longer-term and multi-location validation is still needed.

From the genetic and physiological perspectives, Fernando et al. compared 20 rice genotypes under moderate water limitation during vegetative growth and identified two complementary routes to improved WUE. Tolerant genotypes rely on constitutively low stomatal conductance and enhanced cuticular wax deposition, whereas the adaptive genotypes depends on phenotypic plasticity, including a previously under-recognized enlargement of leaf papillae over stomatal complexes. Carbon isotope analysis confirmed improved intrinsic WUE in the top-performing genotypes, without major yield penalties. These findings align with emerging evidence that higher stomatal density can simultaneously increase photosynthesis and intrinsic WUE under drought in rice (Zhang et al., 2026) and that stomatal behavior remains a central lever for intrinsic WUE across C3 and C4 crops (Ghannoum et al., 2025).

In *Brassica juncea*, Limbalkar et al. evaluated *Brassica carinata*-derived *B. juncea* introgression lines under rainfed and irrigated conditions to identify traits supporting yield stability under moisture-deficit stress. A balanced source–sink ratio and efficient dry-matter partitioning distinguished the best-performing lines under moisture-deficit stress, more clearly than any single trait. Several introgression lines outperformed parental lines for growth rate, harvest index, and reproductive dry-matter accumulation, emerging as promising drought-resilient breeding materials. Balanced source–sink relationships and efficient dry matter partitioning were key determinants of seed yield under rainfed conditions, while several lines emerged as promising drought-resilient breeding materials. A parallel pattern was recently reported in wheat, where the plasticity of source-sink dynamics, rather than a fixed trait, underpins yield stability across fluctuating environments (Wang et al., 2026). Thus, the study highlights interspecific introgression as an effective route for combining drought resilience with yield stability in *B. juncea*.

In addition to these WUE-focused studies, Chandavarapu et al. conducted a high-density genome-wide association study (GWAS) using a United States rice germplasm panel to dissect seedling-stage chilling tolerance. Their study identified 54 quantitative trait loci, including one novel locus, *qNL2-1*, and candidate genes involved in calcium-signaling and brassinosteroid pathways. Because early-season chilling and water-limitation stresses share overlapping signaling components and both threaten seedling establishment, this work broadens the genetic resources available for developing multi-stress-resilient, water-efficient rice cultivars. Integrating these markers with genomic selection (Escamilla et al., 2025), could further accelerate cultivar development by translating QTL discovery into breeding applications.

Together, these five studies provide partial answers to the two questions that motivated this Research Topic. Agronomic and genetic approaches are most effective when management decisions, including irrigation timing, planting density, and nutrient rate, are matched to genotype-specific water-use behavior. The cotton and jujube studies demonstrate this principle through multi-factor optimization frameworks. At the same time, traits that contribute most to WUE are rarely singular; stomatal and cuticular traits, source–sink balance, and stress-signaling pathways interact across scales. Researchers also continue to face the challenge of quantifying plant responses to temperatures above the optimal range, water deficits, and elevated CO_2_ levels, and, more importantly, the interactions among these three factors (Hatfield and Dold, 2019).

## Future frontiers

Several themes within the original scope of this Research Topic remain important frontiers for future investigation. Real-time monitoring of crop water status is advancing rapidly as phenotyping platforms increasingly integrate ground-, drone-, and satellite-based data across spatial and temporal scales (Cudjoe et al., 2026). Precision fertigation and CRISPR-Cas gene editing (Shah, 2026) provide complementary opportunities to improve WUE by linking management innovation with targeted genetic improvement. Integrating the physiological and genetic insights highlighted in this collection with remote sensing, high-throughput phenotyping, and genomic selection could accelerate the development of water-efficient cultivars tailored to site-specific management. Collectively, these studies underscore the value of sustained collaboration among agronomists, physiologists, biotechnologists, engineers, and breeders in advancing the shared goal of producing “more crop per drop.”
